# Sunitinib Indirectly Enhanced Anti-Tumor Cytotoxicity of Cytokine-Induced Killer Cells and CD3^+^CD56^+^ Subset through the Co-Culturing Dendritic Cells

**DOI:** 10.1371/journal.pone.0078980

**Published:** 2013-11-13

**Authors:** Adisak Wongkajornsilp, Valla Wamanuttajinda, Kanda Kasetsinsombat, Sunisa Duangsa-ard, Khanit Sa-ngiamsuntorn, Suradej Hongeng, Kittipong Maneechotesuwan

**Affiliations:** 1 Department of Pharmacology, Faculty of Medicine Siriraj Hospital, Mahidol University, Bangkok, Thailand; 2 Department of Medicine, Faculty of Medicine Siriraj Hospital, Mahidol University, Bangkok, Thailand; 3 Department of Pediatrics, Faculty of Medicine Ramathibodi Hospital, Mahidol University, Bangkok, Thailand; 4 Department of Biochemistry, Faculty of Pharmacy, Mahidol University, Bangkok, Thailand; University of South Alabama Mitchell Cancer Institute, United States of America

## Abstract

Cytokine-induced killer (CIK) cells have reached clinical trials for leukemia and solid tumors. Their anti-tumor cytotoxicity had earlier been shown to be intensified after the co-culture with dendritic cells (DCs). We observed markedly enhanced anti-tumor cytotoxicity activity of CIK cells after the co-culture with sunitinib-pretreated DCs over that of untreated DCs. This cytotoxicity was reliant upon DC modulation by sunitinib because the direct exposure of CIK cells to sunitinib had no significant effect. Sunitinib promoted Th1-inducing and pro-inflammatory phenotypes (IL-12, IFN-γ and IL-6) in DCs at the expense of Th2 inducing phenotype (IL-13) and regulatory phenotype (PD-L1, IDO). Sunitinib-treated DCs subsequently induced the upregulation of Th1 phenotypic markers (IFN-γ and T-bet) and the downregulation of the Th2 signature (GATA-3) and the Th17 marker (RORC) on the CD3^+^CD56^+^ subset of CIK cells. It concluded that sunitinib-pretreated DCs drove the CD3^+^CD56^+^ subset toward Th1 phenotype with increased anti-tumor cytotoxicity.

## Introduction

The mechanisms of tumor immune evasion involve several biological molecules including indoleamine 2, 3-dioxygenase (IDO), PD-L1, GATA and interferon (IFN). IDO, a cytosolic protein that catalyzes the rate-limiting step of tryptophan (Trp) metabolism, stimulates immune tolerance in human cancer [1]. IDO generates immunosuppressive dendritic cells (DCs) [2]. Trp metabolites mediate cytotoxic effects on CD8^+^ tumor-infiltrating lymphocytes and CD4^+^Th1 cells [3]–[5]. PD-L1 can have an inhibitory function that primarily acts to inhibit the priming and activation of immune responses and T cell-mediated killing of cancer cells in particular in the tumor beds [6]. The zinc finger DNA binding GATA factors coordinate cellular maturation with proliferation arrest and cell survival [7]. Alteration of GATA factors was shown to be causatively involved in various cancers in human patients [7]. GATA-3 primarily induces Th2 differentiation [8] and therefore causes Th2 immune deviation that leads to the expansion of fibrocytes with immunosuppressive properties observed in patients with cancer [9]. This may be the mechanism that GATA-3 contributes to tumor progression via immune evasion. The above data suggested the requirement of therapeutic overriding of tumor immune evasion by boosting cytotoxic effects of responsible effector cells.

Cytokine-induced killer (CIK) cells have been deployed against a number of solid tumors with *in vitro* and *in vivo* evidences. The major effector of CIK cells is the CD3^+^CD56^+^ subset [10], [11]. The anti-tumor action of CIK cells could be augmented after being co-cultured with dendritic cells (DCs)[12]–[15]. The depletion of regulatory T cell (Treg) subset in CIK cells after the co-culture with DCs was proposed as the responsible mechanism [13]. We previously observed similar enhancement of the anti-tumor action of the isolated CD3^+^CD56^+^ subset against cholangiocarcinoma [16] and osteosarcoma [17] after being co-cultured with DCs. This observation implied that the activity of CD3^+^CD56^+^ subset was not invariably naturally active, but inducible. The *ex vivo* optimization for the anti-tumor activity of the CD3^+^CD56^+^ subset as well as the dissection for the involved signal transduction has posed as a challenge for CIK cell-based immunotherapy. We approached this challenge through the treatment of CIK cells, co-cultured DCs with a promising molecule, sunitinib.

Sunitinib, a protein kinase inhibitor (PKI), is conventionally intended for direct treatment of lung cancer and renal cell carcinoma. It indirectly affects the tumors through the host components of immune response [18]. The pharmacological concentrations of sunitinib had no effect toward PI3K and ERK phosphorylation in NK cells and did not exert any toxicity toward peripheral blood mononuclear (PBMCs) [19]. Not all tyrosine kinase inhibitors provide the beneficial effects toward immune cells [18]. Only sunitinib could enhance the maturation and the expansion of DCs. Unlike sunitinib, sorafenib at therapeutic concentrations induced human NK cell-derived cytotoxic activity, IFN-γ release [19], suppressed mouse DCs and antigen-specific T cells functions [20].

Sunitinib might exert its immunostimulatory activity through the modulation of the ratio of immunostimulatory versus immunoregulatory cells. Recently sunitinib was shown to reverse the immune suppression of tumor microenvironment (TME) by suppressing the development of regulatory T cells (Treg) [21]. Both Treg and myeloid-derived suppressor cells (MDSC) are the major immunosuppressive cellular components in TME [22], [23]. The presence of Treg subset compromised the overall anti-tumor activity of CIK cells [16], [17], [24]. The fraction of peripheral blood MDSC [25], [26] and Treg [25], [27], [28] were dramatically decreased in subjects treated with sunitinib. In contrast, the fraction of DCs was significantly increased after sunitinib treatment and this correlated with tumor regression in patients with renal cell carcinoma [26]. The combination of sunitinib treatment with DC vaccination acted synergistically in suppressing the implanted melanoma in mice [29]. The responders with tumor regression after sunitinib treatment were associated with the reduction in MDSC and Treg in the TME in concomitant with the rising of CD8^+^ T cells. Sunitinib shifted tumor-infiltrating lymphocytes (TILs) in mice from releasing Th2 cytokines (IL-10, TGF-β) to Th1 cytokines (IFN-γ). The expression of co-inhibitory molecules (CTLA-4 and PD-1) and Foxp3 in these TILs was also suppressed. This reversal of immunosuppression was proposed to be mediated through the inhibition of c-kit in MDSCs [30]. The suppressive activity of sunitinib on MDSC might be counteracted by GM-CSF-enriched microenvironment [31]. The immunomodulation might be mediated through anti-VEGFR and NF-κB-suppressive actions of sunitinib. The heightened proliferation and antigen-specific T-cell activity of CD8^+^ T cells was attributed to the suppression of STAT3 [32]. However, other investigators reported the absence of favorable immunological action of sunitinib. Sunitinib was unable to reverse VEGF/tumor supernatant-induced suppression of DC maturation [33]. Some renal cell carcinoma subjects treated with sunitinib for 4 weeks carried lower Th1/Th2 ratio in peripheral blood [34], as opposed to those found in the earlier studies [27].

Improving the density as well as the activity of CD3^+^CD56^+^ subset, while suppressing those of Treg subset, would be desirable for CIK cell-based immunotherapy. We investigated whether the introduction of sunitinib to the DC-CIK co-culture system could improve anti-tumor effects. We examined the alteration in the proportion of CIK cell subsets. For qualitative change, we measured the alteration in the maturation status or immunological markers of both DCs and CIK cells. In DCs, the markers studied included Th1 promoting genes (IL-12, IFN-γ); Th2 promoting gene (IL-13); Th17 promoting genes (IL-23, IL-6); and Treg promoting genes (PD-L1, IDO, IL-10). In CIK cells, the markers assessed included Th1 genes (IFN-γ, T-bet); Th2 genes (IL-4, GATA-3); Th17 genes (RORC, IL-17, STAT3); and Treg (IDO, IL-10).

## Materials and Methods

### Ethic Statement

All subjects understood and signed the informed consent form before the participation. The study protocol with the accompanying informed consent form conforms to the ethical guidelines of the 1975 Declaration of Helsinki and was approved by the Institutional Review Board of the Faculty of Medicine Siriraj Hospital, Mahidol University.

### Generation of CIK Cells and DCs from Peripheral Blood Mononuclear Cells

CIK cells and DCs were generated from PBMCs of 6 consented healthy volunteers. PBMCs were isolated from whole blood by Ficoll gradient centrifugation (IsoPrep, Robbins Scientific, Sunnyvale, CA). The cells were allowed to adhere over the 6-well plate at a density of 1.2×10^6^ cells/mL/well for 1 h at 37°C in RPMI 1640, 10% FBS, 100 U/mL penicillin, and 100 µg/mL streptomycin. The adherent cells (5.0×10^4^ cells/well) were used to generate DCs.

To generate CIK cells, non-adherent PBMCs were resuspended in RPMI 1640 (Invitrogen, Carlsbad, CA), 10% FBS, 25 mM Hepes, 100 U/mL penicillin and 100 µg/mL streptomycin. Human interferon γ (IFN-γ, 1,000 U/mL (Amoytop Biotech, Xiamen, China) was added and incubated at 37°C, 5% CO_2_ for 24 h. After 24-h incubation, 50 ng/mL monoclonal antibody against CD3 (eBioscience, San Diego, CA)and 300 IU/mL IL-2 (Amoytop Biotech) were added. CIK cells were maintained at a density of ≤6×10^6^ cells/mL in RPMI 1640, 10% FBS, 300 IU/mL IL-2, 25 mM Hepes, 2 mM L-glutamine, 100 U/mL penicillin, and 100 µg/mL streptomycin with medium replacement every 5 days. Cells were harvested on day 14 with apparent viability above 90%.

To generate DCs, the adherent cells were maintained in 2 mL RPMI 1640, 10% FBS, 400 U/mL granulocyte-macrophage colony-stimulating factor (GM-CSF, Amoytop Biotech), 500 U/mL IL-4 (Amoytop Biotech) for 14 d. DC maturation could be achieved by adding 1,000 U/mL tumor necrosis factor α (TNF-α; Amoytop Biotech) in the final 24 h. Some designated wells were treated with 1 µM sunitinib (Sigma, St. Louis, MO) for 48 h. The viability of mature DCs was above 95%.

To generate macrophages, the adherent cells were maintained in 2 mL RPMI 1640, 10% FBS, 400 U/mL granulocyte-macrophage colony-stimulating factor (GM-CSF, Amoytop Biotech) for 7 d.

### Preparation of CD3^+^CD56^+^ Cells

An aliquot of CIK cells (1.0×10^8^ cells) on day 14 was purified for CD3^+^CD56^+^ subset using CD3 Microbeads kit and CD56 Microbeads kit (Miltenyi Biotec, Germany) according to the manufacturer instruction. This usually yielded 0.8–2.0×10^7^ purified CD3^+^CD56^+^ cells.

### Co-culture of CIK Cells and DCs

On day 14 after CIK and DCs generation, CIK cells or the purified CD3^+^CD56^+^ cells were seeded on DCs of different conditions at the stimulators (DCs) : responders (CIK cells)(S:R) ratio of 1∶20. The co-cultured cells were maintained in RPMI 1640, 10% FBS, 25 mM Hepes, 2 mM L-glutamine, 100 U/mL penicillin, and 100 µg/mL streptomycin, 300 IU/mL IL-2 for 5 days prior to *in vitro* cytotoxicity assay.

### Primary Cultured Cholangiocarcinoma Cells Isolated from Sediments of Biliary Fluid

A human cholangiocarcinoma cell line prepared from intrahepatic biliary fluid, HubCCA1 [16], was propagated in growth medium (DMEM, 15% FBS, 1 mM sodium pyruvate, 1 mg/mL insulin, 0.66 mg/mL transferrin, 0.67 µg/mL sodium selenite, 0.1 mM non-essential amino acid solution, 2 mM L-glutamine, 50 unit/mL penicillin, and 50 µg/mL streptomycin) at 37°C with 5% CO_2_.

### Fluorescence-activated Cell Sorting (FACS) Analysis

Either DCs or CIK cells were washed twice in PBS containing 5% FBS (PBS/FBS) and resuspended in 100 µL PBS/FBS. The cell pellet was incubated with 2 µL of the corresponding primary monoclonal antibodies (1 mg/mL) for 30 min at 25°C, washed twice and resuspended in 200 µL of PBS/FBS. For the staining of intracellular immunogens, cells were fixed and permeabilized prior to the intracellular staining in accordance with the manufacturers. Flow cytometry analysis on 10,000 cells was performed using a FACSCalibur (Becton Dickinson, San Jose, CA). The employed primary mouse monoclonal antibodies raised against human immunogens included anti-FOXP3-Alexa Fluor 488, anti-CD4-PE-Cy5, anti-CD25-PE from Biolegend, anti-IL-10-Alexa Fluor 647, anti-CD3-FITC, anti-CD56-PE, anti-CD80-FITC, anti-CD83-PE, anti-CD86-PE-Cy5, anti-CD40-APC from eBiosceince, anti-RORC-PerCP, anti-IL-17-APC from R&D Systems. Data were analyzed using FlowJo version 10.0.5.

### Cytotoxic Assay

Propidium iodide (PI)-based cytotoxic assay was used to estimate the anti-tumor cytotoxic activity of CIK cells. The tumor cells (5×10^3^ cells/well) were seeded as target cells on the 96-well plate for 24 h at 37°C, 5% CO_2_. The target cells were washed with serum-free RPMI and co-cultured with the effector cells at the designated effector to target (E:T) ratio in 80 µL RPMI/well for 4 h at 37°C, 5% CO_2_. For IFN-γ neutralization, 0.02 µg/mL anti-IFN-γ (clone 25718, R&D Systems) was added to the effector cells 2 h prior to the co-culture. PI (20 µL of 10 µg/mL in PBS) was added to and incubated with the cell mixture for additional 30 min. The mixture was measured for fluorescence with an excitation wavelength of 482 nm and an emission wavelength of 630 nm using the SpectraMax M5 microplate reader (Molecular Devices, Sunnyvale, CA). The background wells were those with the corresponding numbers of effector cells, but without target cells. The 100% lysis came from wells containing target in RPMI cells plus 20 µL isopropanol. The 0% lysis came from wells containing only the target cells. The % cytotoxicity was calculated using the following expression (see in Figure S1):
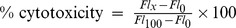



*Fl*_0_ represents the fluorescence of the well containing the target cells without the exposure to any effector cells. *Fl*_100_ represents the fluorescence of from the well containing the target cells in RPMI plus 20% isopropanol. *Fl_x_* represents the fluorescence of the well containing the target cells after the exposure to the indicated numbers of effector cells.

### RNA Preparation and Quantitative Real-time PCR Analysis

Total RNA was extracted from different conditions of CD3^+^CD56^+^ cells, macrophages, iDCs and mDCs. Cells were homogenized in 350 µL of RA1 buffer and 3.5 µL of β-mercaptoethanol (illustra™ RNAspin Mini RNA Isolation Kit, GE Healthcare, UK) to isolate total RNA. Reverse transcription was performed with 1 µg of total RNA. First-strand cDNA synthesis was performed with the ImProm-II Reverse Transcription System (Promega, Madison, WI). The gene-specific primers pairs (Table 1) were designed using Primer Express 3.0 (ABI, Foster City, CA) and ordered from 1st BASE (Singapore). They were amplified using FastStart SYBR® Green Master *(*Roche Diagnostics, Mannheim, Germany) and StepOnePlus Real*-*Time PCR system (ABI). Real-time PCR was performed using 1.5 µL of 200 ng/µL cDNA in 15 µL reaction mixture with the following conditions: 95°C for 10 min, followed by 40 cycles of amplification at 95°C for 15 sec, 60°C for 40 sec, and 72°C for 40 sec. The obtained Ct’s were subtracted with the Ct of GAPDH of the same condition to obtain ΔCt. The ΔCt’s of the treated cells were subtracted with ΔCt’s of the untreated cells of the same period to obtain ΔΔCt’s. The fold-changes could be obtained from the expression of 2^−ΔΔCt^.

**Table 1:** The primer pairs for real-time RT-PCR.

Genes	Oligonucleotides (5′→3′)	Size (bp)	Annealing (°C)
GAPDH	Forward: GAAATCCCATCACCATCTTCC	124	60
	Reverse: AAATGAGCCCCAGCCTTCTC		
PD-L1	Forward: TCAATGCCCCATACAACAAA	120	60
	Reverse: TGCTTGTCCAGATGACTTCG		
IDO	Forward: AGTCCGTGAGTTTGTCCTTTCAA	68	60
	Reverse: TTTCACACAGGCGTCATAAGCT		
GATA-3	Forward: ACTACGGAAACTCGGTCAGG	100	60
	Reverse: CAGGGTAGGGATCCATGAAG		
IFNγ	Forward: GTGTGGAGACCATCAAGGAAGAC	80	60
	Reverse: CAGCTTTTCGAAGTCATCTCGTTT		
IL-4	Forward: AACAGCCTCACAGAGCAGAAGAC	101	60
	Reverse: GCCCTGCAGAAGGTTTCCTT		
IL-6	Forward: GCTGCAGGCACAGAACCA	68	60
	Reverse: ACTCCTTAAAGCTGCGCAGAA		
IL-10	Forward: CTGGGTTGCCAAGCCTTGT	100	60
	Reverse: AGTTCACATGCGCCTTGATG		
IL-12	Forward: GCAAAACCCTGACCATCCAA	100	60
	Reverse: TGAAGCAGCAGGAGCGAAT		
IL-13	Forward: GAGTGTGTTTGTCACCGTTG	253	60
	Reverse: TACTCGTTGGTCGAGAGCTG		
IL-23	Forward: GCTTACAAACTCGGTGAACAACTG	80	60
	Reverse: TCCACTTGCTTTGAGCCTGAT		
RORC	Forward: CCACAGAGACATCACCGAGCC	114	60
	Reverse: GTGGATCCCAGATGACTTGTCC		
STAT3	Forward: ACCAAGCGAGGACTGAGCAT	90	58
	Reverse: TGTGATCTGACACCCTGAATAATTC		
TGF-β	Forward: GCGTGCTAATGGTGGAAACC	100	60
	Reverse: GCTTCTCGGAGCTCTGATGTGT		
T-bet	Forward: AGGATTCCGGGAGAACTTTGA	123	60
	Reverse: TACTGGTTGGGTAGGAGAGGAGAGTA		

### Statistical Analysis

The results are shown as mean ± standard error of the mean (SEM) of triplicate determinants. Data were plotted using GraphPad Prism version 5.03. Two-way ANOVA was used to determine the significance of difference between means of cytotoxic experiments. Student’s *t*-test was used for real-time PCR analysis. A *p* value of less than 0.05 was considered significant.

## Results

### The Cytotoxic Activity of CIK Cells after the Priming with Sunitinib-treated DCs toward Cholangiocarcinoma Cell Line

**Figure 1:**
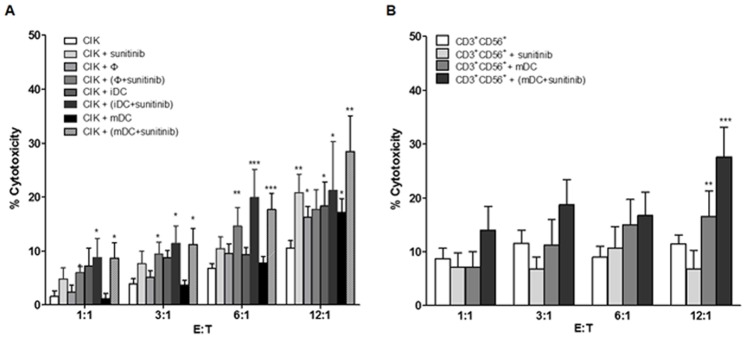
The cytotoxic activity against HubCCA1 cell line after the priming with sunitinib-pretreated DCs. The CIK cells (A) at 1.0×10^5^ cells/well from each condition were incubated with the attached HubCCA1 cells (5,000 cells/well) for 4 h before the PI assay. The CIK cell preparations comprised untreated condition, direct sunitinib treatment, macrophage co-culture, sunitinib-treated macrophage co-culture, iDC co-culture, and sunitinib-treated iDC co-culture, mDC co-culture, and sunitinib-treated mDC co-culture. The isolated CD3^+^CD56^+^ cells (B) were studied in similar fashion. These included untreated CD3^+^CD56^+^ cells, direct sunitinib treatment, mDC co-culture, and sunitinib-treated mDC co-culture. * and ** designate data with significant different from those of the untreated CIK cells at the same effector to target (E:T) ratio with p<0.05 and <0.01 respectively.

Among all investigated conditions of effector cells, the untreated CIK cells provided the lowest cytotoxicity toward the HubCCA-1 (Fig. 1A). The cytotoxicity was improved after the co-culture with mDC. The highest anti-tumor cytotoxic activity came from CIK cells that had been co-cultured with either sunitinib-treated iDCs or sunitinib-treated mDCs. This enhancement could not be obtained from CIK cells co-cultured with sunitinib-treated macrophages. The direct exposure of CIK cells to sunitinib could not confer any significant improvement in the anti-tumor cytotoxicity over that of the untreated CIK cells until the E:T ratio reached 12∶1, and therefore demonstrated little enhancement. Isolated CD3^+^CD56^+^ subset contained anti-tumor cytotoxic activity (Fig. 1B). Likewise, the direct exposure of CD3^+^CD56^+^ subset to sunitinib could not confer further significant improvement. The co-culture of CD3^+^CD56^+^ subset with sunitinib-treated mDCs provided the optimal improvement.

### The Alteration in the Polarization of Macrophages, iDCs and mDCs after the Exposure to Sunitinib

**Figure 2:**
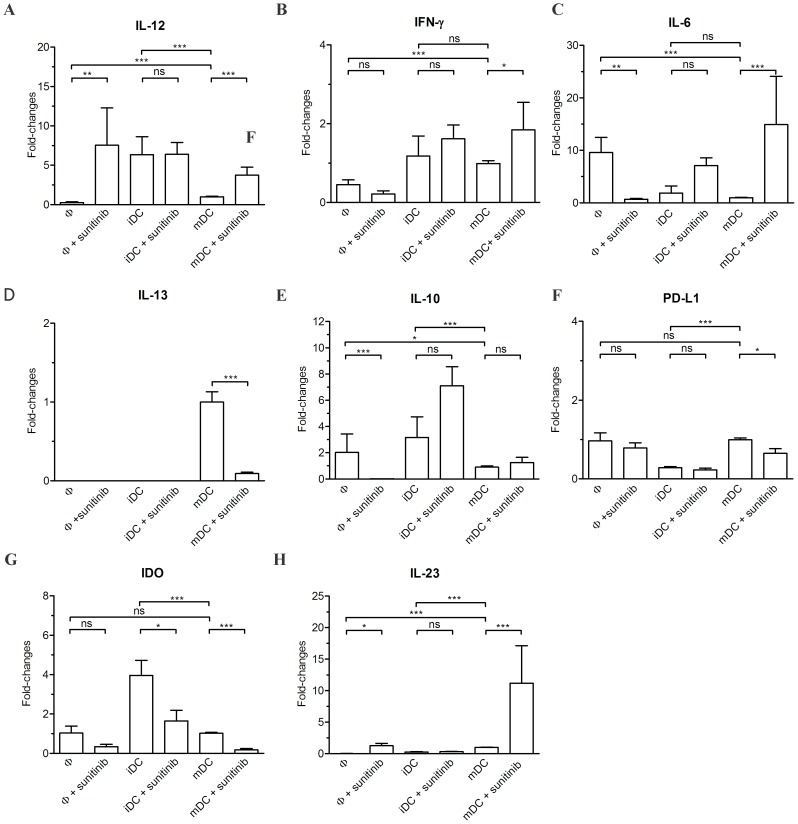
The real-time RT-PCR analysis in macrophages (Φ), iDC and mDC after sunitinib exposure. These cells were evaluated for the expression of IL-12 (A), IFN-γ (B), IL-6 (C), IL-13 (D), IL-10 (E), PD-L1 (F), IDO (G), and IL-23 (H). The expression levels of these genes were normalized with those of their respective untreated mDCs.

Macrophages, iDCs and mDCs were studied for their polarization using real-time RT-PCR analysis for a number of markers (Table 1). The untreated macrophages contained lower IL-12 expression (Fig. 2A) than those in iDCs and mDCs. After sunitinib treatment, the level of IL-12 expression was enhanced in macrophages and mDCs, but not in iDCs. Both iDCs and mDCs carried higher IFN-γ expression than did macrophages. Only in mDCs was the expression of IFN-γ (Fig. 2B) significantly increased after sunitinib treatment. The expression level of IL-6 (Fig. 2C) was rising in mDCs after sunitinib treatment, but was reciprocally suppressed in macrophages. The expression of IL-13 in macrophages and iDCs, but not mDCs, was undetectable. The IL-13 expression in mDCs was suppressed after sunitinib treatment (Fig. 2D). The untreated macrophages and iDCs contained higher IL-10 expression (Fig. 2E) than did mDCs. Upon sunitinib treatment, the expression of IL-10 was decreased in macrophages, but not significantly altered in iDCs nor mDCs. Sunitinib treatment suppressed the expression of PD-L1 (Fig. 2F) in mDCs. The IDO expression (Fig. 2G) was suppressed in iDCs and mDCs after sunitinib treatment. Sunitinib enhanced the expression of IL-23 (Fig. 2H) in macrophages and mDCs, but not in iDCs.

### The Alteration in the Maturity of Macrophages, iDCs, and mDCs after Sunitinib Treatment

**Figure 3:**
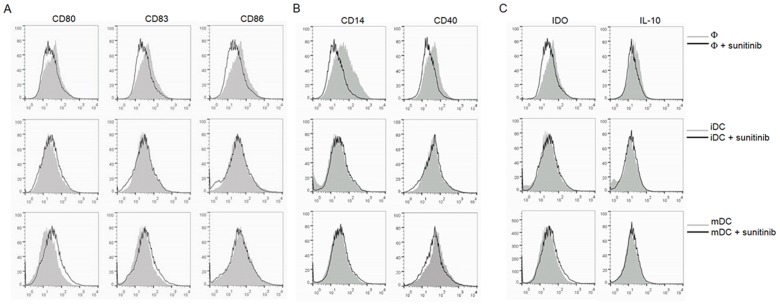
The FACS analysis for the maturity of macrophages, iDCs, and mDCs after sunitinib exposure. The markers for the maturity of DCs (A) are CD80, CD83 and CD86. The macrophage markers (B) are CD14 and CD40. The selected immunosuppressive molecules (C) are IDO and IL-10.

Sunitinib treatment did not promote DC maturation as demonstrated by the unchanged expression of CD80, CD83 and CD86 (Fig. 3A). In contrast, sunitinib-treated macrophages revealed not only less DC maturation markers (CD80, CD83, CD86 and CD40), but also less macrophage markers (CD14 and CD40, Fig. 3B). There was no alteration in IL-10, and IDO (Fig. 3C) in sunitinib-treated iDCs and -treated mDCs, whereas the IDO expression was decreased in sunitinib-treated macrophages.

### The Examination for the Alteration of the Ratios of CD3^+^CD56^+^, Treg, and Th17 Subsets in the Whole CIK Cell Population after the Priming with Sunitinib-treated DCs

**Figure 4:**
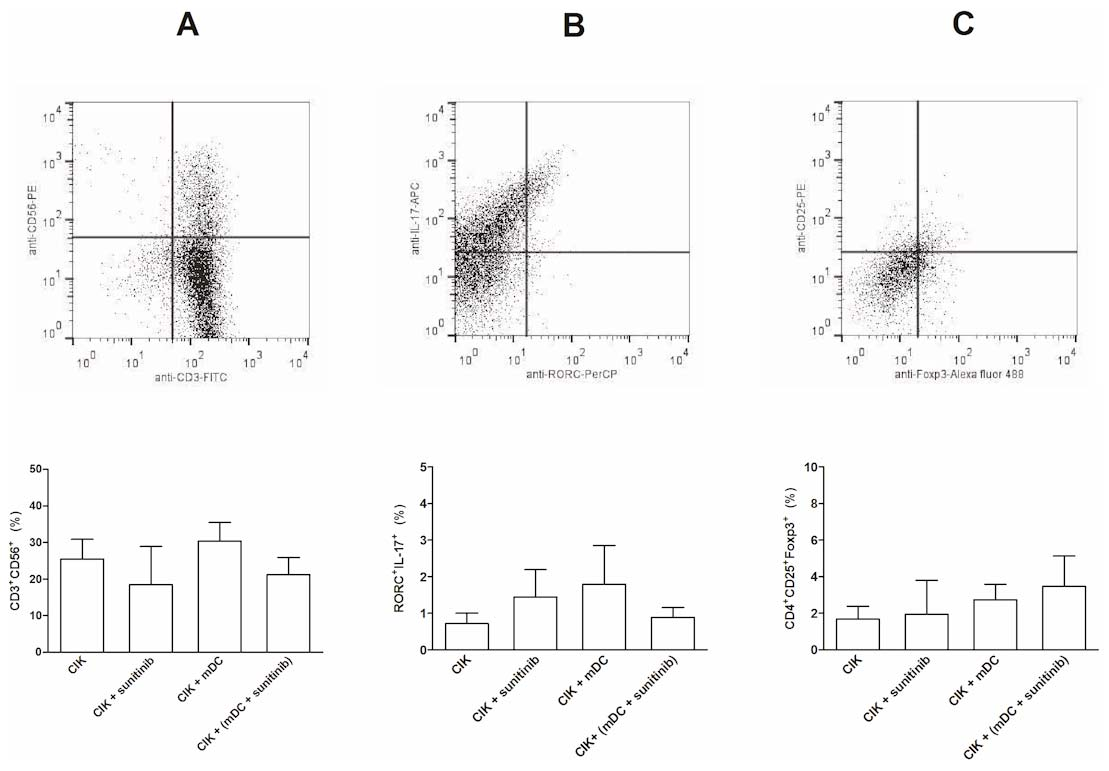
The FACS analysis for the alteration in the proportion of subpopulations in CIK cells. The studied subpopulations included CD3^+^CD56^+^(A), Th17 (RORC^+^IL-17^+^, B) and Treg (CD4^+^CD25^+^Foxp3^+^, C) subsets. The corresponding dot plot analysis demonstrated the gating of each subset. The CIK cells were either exposed to sunitinib directly, primed with mDCs or primed with sunitinib-pretreated DCs.

CIK that had been either directly treated with sunitinib, primed with mDCs or primed with sunitinib-pretreated mDCs did not significantly alter the proportions of the CD3^+^CD56^+^ subset (Fig. 4A), the Th17 subset (Fig. 4B), nor the Treg (CD4^+^CD25^+^Foxp3^+^) subset (Fig. 4C).

### The Analysis for the Polarization of CD3^+^CD56^+^ Cells after the Priming with Either Sunitinib-treated mDCs or Untreated mDCs

**Figure 5:**
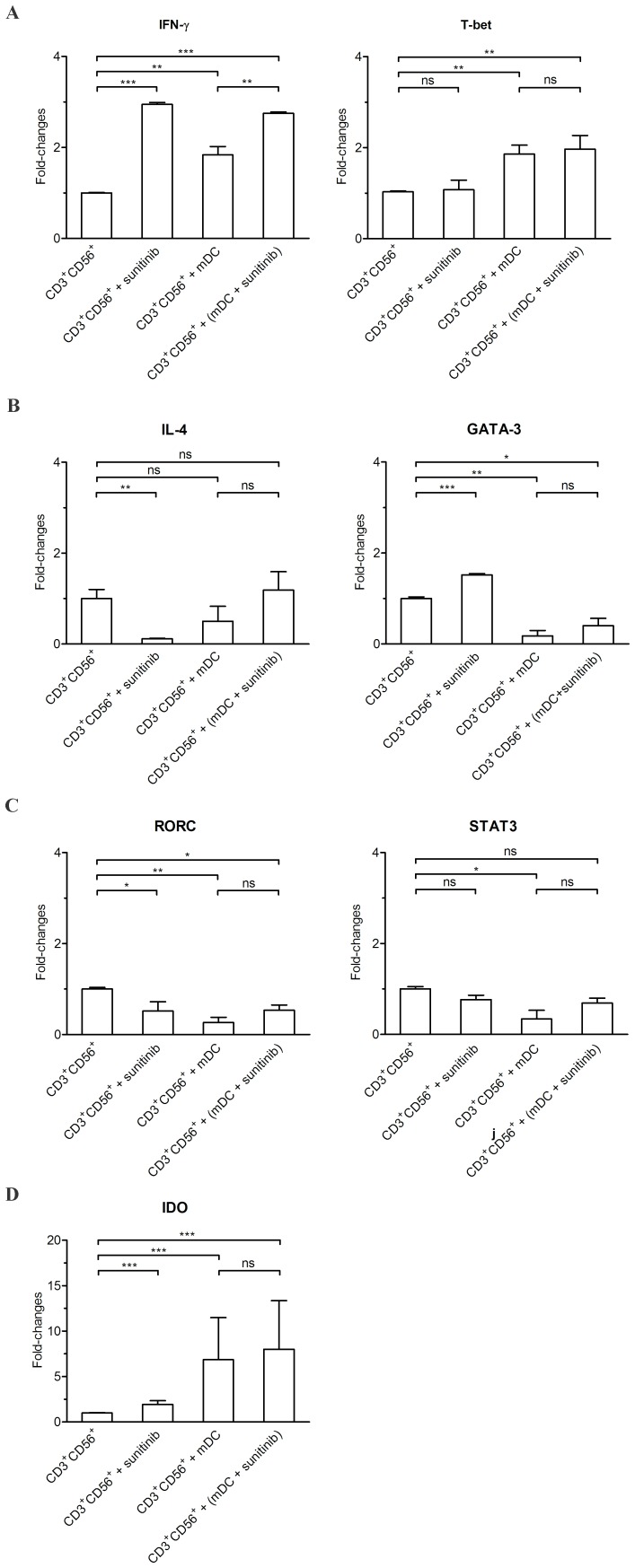
The real-time RT-PCR analysis for the polarization of CD3^+^CD56^+^ subset after different treatments. The CD3^+^CD56^+^ subset that had been directly exposed to sunitinib or co-cultured with sunitinib-pretreated mDCs were analyzed for the expression of IFN-γ, T-bet, IL-4, GATA-3, RORC, STAT3, and IDO.

The co-culture of CD3^+^CD56^+^ cells with untreated mDCs raised the expression of IDO, and Th1 markers (IFN-γ and T-bet) (Fig. 5). In contrast, the expression of Th2 markers (GATA-3) and Th17 (RORC, STAT3) markers was reduced. The co-culture of CD3^+^CD56^+^ cells with sunitinib-pretreated mDCs maintained the rising IDO, IFN-γ, T-bet; the lessening of GATA-3 and RORC.

### The Cytotoxic Activity of all Treatment Conditions of CD3^+^CD56^+^ Cells Required IFN-γ

**Figure 6:**
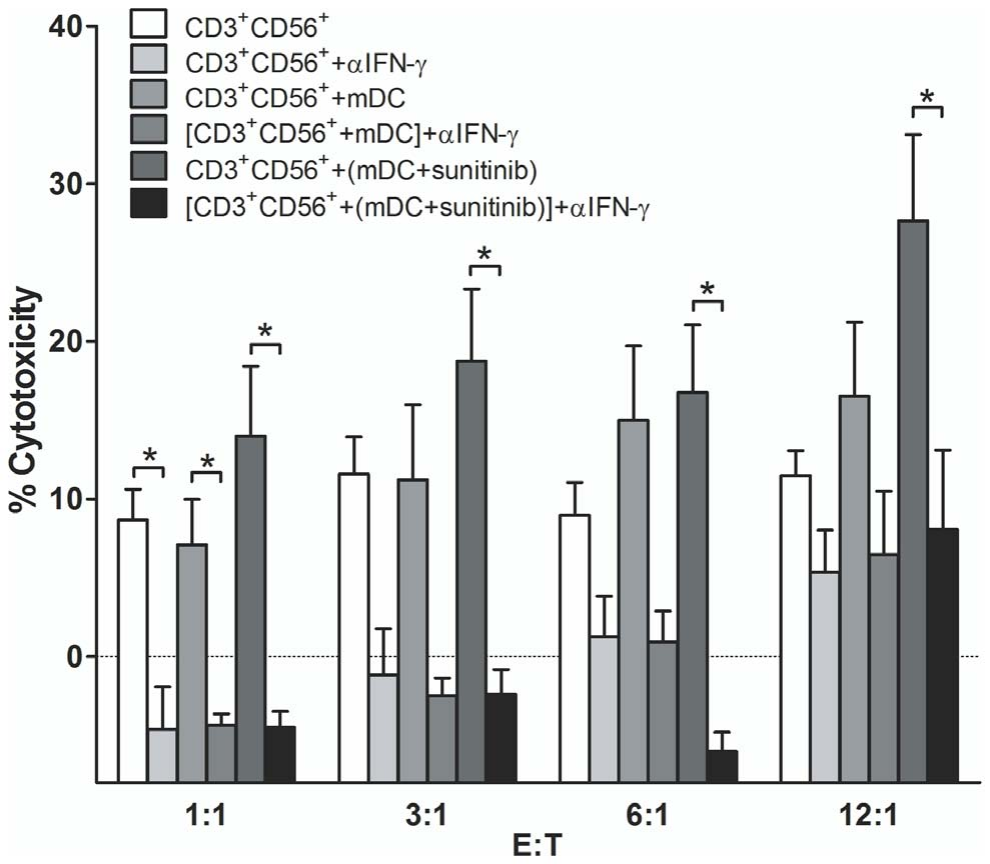
The cytotoxic activity of all conditions of CD3^+^CD56^+^ cells could be neutralized with αIFN-γ treatment. The CD3^+^CD56^+^ cells from each condition were incubated with the attached HubCCA1 cells (5,000 cells/well) for 4 h before the PI assay. These conditions included the untreated CD3^+^CD56^+^ cells, αIFN-γ treatment to CD3^+^CD56^+^ cells, CD3^+^CD56^+^ cells primed with mDC, αIFN-γ treatment to CD3^+^CD56^+^ cells primed with mDC, CD3^+^CD56^+^ cells primed with sunitinib-pretreated mDC, and αIFN-γ treatment to CD3^+^CD56^+^ cells primed with sunitinib-pretreated mDC. * designates conditions that provided statistically difference after αIFN-γ treatment at the same E:T ratio with p<0.05.

The CD3^+^CD56^+^ subset that had been exposed to mDC or sunitinib treated mDCs were examined whether they mediated their anti-tumor cytotoxic action through IFN-γ. The isolated CD3^+^CD56^+^ subset from each condition was pretreated with the neutralizing monoclonal anti-IFN-γ (αIFN-γ) prior to the exposure to the target HubCCA1 target cells. All studied conditions of CD3^+^CD56^+^ subset were susceptible to the suppressive effect of anti-IFN-γ (Fig. 6).

## Discussion

To our knowledge, the present finding provided the first notion that the CD3^+^CD56^+^ subset of CIK cells was not invariably naturally active. The CD3^+^CD56^+^ subset could be polarized toward either Th1 or Th2 phenotype that in turn shapes its anti-tumor activity. The resting CD3^+^CD56^+^ subset predominantly expressed Th2 phenotypes, but shifted to Th1 phenotypes upon the exposure to sunitinib-pretreated DCs. The induction of Th1 immune response was first observed in T cells isolated from subjects administered with sunitinib [27]. We investigated further whether the Th1-promoting action of sunitinib was derived directly from CIK cells or indirectly from mDCs. Our data revealed the sequential phenotypic changes in DCs after exposure to sunitinib. Our observation confirmed and expanded the previous report that CD3^+^CD56^+^ cells could have their anti-tumor activity expanded after the exposure to mDCs [16] and sunitinib-pretreated mDCs further enhanced this activity. This enhancement could not be simply introduced through the direct exposure of CD3^+^CD56^+^ cells to sunitinib.

### Modulation of DCs and Macrophages by Sunitinib

Since the improvement in anti-tumor cytotoxicity of CIK cells resulted mainly from the exposure to sunitinib-pretreated mDCs, we hypothesized that the phenotypic alterations in DCs from sunitinib might be one of the underlying mechanisms. It was possible that sunitinib could induce DC maturation that led to the corresponding enhancement of the anti-tumor cytotoxicity of CIK cells. Using the staining for the DC maturation markers (CD80, CD83 and CD86), apparently there was no significant change in DC maturation in agreement with an earlier study [33]. The expression of Th1-polarizing cytokines (IL-12, IFN-γ and IL-6) was enhanced, whereas the expression of Th2-polarizing cytokine (IL-13) and the regulatory phenotype (PD-L1, IDO) were suppressed in sunitinib-treated mDCs. The increasing IL-23 expression in sunitinib-treated mDCs should foster the conversion of the nearby Treg toward Th17 cells. The sunitinib-treated monocyte-derived macrophages carried lessen DC maturation markers as well as lessen M1 differentiation markers (CD14 and CD40). The M2 differentiation was also suppressed as evidenced by the lowering IL-10 expression that might favor anti-tumor action. Taken together, sunitinib shifted mDCs toward Th1-polarizing phenotype and away from both Th2-polarizing and regulatory phenotypes.

### Sunitinib-pretreated DCs Drove CD3^+^CD56^+^ Cells toward Th1 Polarization

Following the phenotypic change in sunitinib-treated mDCs, we investigated whether there were subsequent alterations in the proportion or the phenotypes of the co-culturing CIK subsets. There was no significant alteration in the proportion of any subset with any treatment. As opposed to the earlier study [13], we could not observe the lessening of Treg subset following the co-culture with DCs. The quantitative change was evaluated through the alteration in the proportion of CD3^+^CD56^+^ subset within the whole CIK cell population. The CD3^+^CD56^+^ subset proportion was not significantly altered after the exposure to sunitinib-pre-treated mDCs. The functional change was observed through the monitoring for alterations in Th1/Th2/Th17 phenotypes. The polarization toward Th1 differentiation of the CD3^+^CD56^+^ subset was evidenced by the heightening expression of IFN-γ and T-bet. The Th2 differentiation were lessened as evidenced by decreasing GATA3 expression. The alteration in Th1/Th2 phenotypes was in agreement with the observations in DCs. The Th17 differentiation in CD3^+^CD56^+^ subset disagreed with the observation in DCs since RORC expression was decreased while STAT3 expression was not significantly altered. This observation was not unexpected, since the Treg subset, not the CD3^+^CD56^+^ subset, was envisaged to undergo Th17 differentiation [35]. The heightening antitumor activity in all conditions of CD3^+^CD56^+^ subset relied heavily on IFN-γ secretion as this anti-tumor action could be reverse with the neutralizing anti-IFN-γ mAb.

Earlier sunitinib study involving mDCs reported the increasing frequency of mDCs in subjects receiving systemic sunitinib treatment [26] with no deleterious effect toward their immunostimulatory function. We have characterized the changes in sunitinib-pretreated mDCs as well as the co-culturing CIK cells regarding to the Th1/Th2/Treg balance. Our *ex vivo* observation implied the immunostimulatory action of sunitinib in addition to the elimination of immunoregulatory cells as reported by others [18], [20], [21]. Although our studied sunitinib concentration (1 µM) was beyond pharmaceutical concentration, we did not observe any deleterious effect toward mDCs in agreement with the earlier study [20]. The employed concentration was well above the recommended trough plasma concentration (Cmin) at 94 nM [36] and the observed maximal plasma concentration (Cmax) at 188 nM [37], signified the application of *ex vivo* approach to circumvent systemic adverse reactions [38] in clinical trials. The future direction for CIK cell-based immunotherapy may aim to raise the Th1 phenotype of its effectors in addition to neutralizing the immunosuppressive activity in its Treg subset or in TME.

## Supporting Information

Figure S1 (TIF)
